# Effects of a Semisynthetic Catechin on Phosphatidylglycerol Membranes: A Mixed Experimental and Simulation Study

**DOI:** 10.3390/molecules28010422

**Published:** 2023-01-03

**Authors:** Elisa Aranda, José A. Teruel, Antonio Ortiz, María Dolores Pérez-Cárceles, José N. Rodríguez-López, Francisco J. Aranda

**Affiliations:** 1 Departamento de Bioquímica y Biología Molecular-A, Facultad de Veterinaria, Universidad de Murcia, 30100 Murcia, Spain; 2 Departamento de Medicina Legal y Forense, Facultad de Medicina, Instituto de Investigación Biomédica (IMIB-Arrixaca), Universidad de Murcia, 30120 Murcia, Spain

**Keywords:** catechin, dimyristoylphosphatidylglycerol, DSC, FTIR, X-ray diffraction, molecular dynamics

## Abstract

Catechins have been shown to display a great variety of biological activities, prominent among them are their chemo preventive and chemotherapeutic properties against several types of cancer. The amphiphilic nature of catechins points to the membrane as a potential target for their actions. 3,4,5-Trimethoxybenzoate of catechin (TMBC) is a modified structural analog of catechin that shows significant antiproliferative activity against melanoma and breast cancer cells. Phosphatidylglycerol is an anionic membrane phospholipid with important physical and biochemical characteristics that make it biologically relevant. In addition, phosphatidylglycerol is a preeminent component of bacterial membranes. Using biomimetic membranes, we examined the effects of TMBC on the structural and dynamic properties of phosphatidylglycerol bilayers by means of biophysical techniques such as differential scanning calorimetry, X-ray diffraction and infrared spectroscopy, together with an analysis through molecular dynamics simulation. We found that TMBC perturbs the thermotropic gel to liquid-crystalline phase transition and promotes immiscibility in both phospholipid phases. The modified catechin decreases the thickness of the bilayer and is able to form hydrogen bonds with the carbonyl groups of the phospholipid. Experimental data support the simulated data that locate TMBC as mostly forming clusters in the middle region of each monolayer approaching the carbonyl moiety of the phospholipid. The presence of TMBC modifies the structural and dynamic properties of the phosphatidylglycerol bilayer. The decrease in membrane thickness and the change of the hydrogen bonding pattern in the interfacial region of the bilayer elicited by the catechin might contribute to the alteration of the events taking place in the membrane and might help to understand the mechanism of action of the diverse effects displayed by catechins.

## 1. Introduction

The number of healthful effects attributed to green tea catechins is remarkable. The health promoting properties of catechins include protection from inflammatory and neurodegenerative diseases, obesity, metabolic syndrome, diabetes, and hypertension [1,2]. In addition, different studies have provided evidence that catechins display antimicrobial effects on both Gram-positive and Gram-negative bacteria [3], and that they impair the infectivity of a series of both animal and human viruses [4].

A most outstanding characteristic of catechins is that they show both chemo-preventive and chemotherapeutic activities against a great variety of different cancers [5]. The exceptional anticancer activity of catechins is exerted by modulating different hallmarks and suppressing characteristics of cancer, including sustaining proliferative signals, evading growth suppressors, avoiding immune destruction, inducing angiogenesis, resisting cell death, and tumor promoting inflammation [6,7]. Almost all of these anticancer achievements are fulfilled essentially as a result of the interaction between catechins and a variety of intracellular targets, membrane proteins, and the plasma membrane [8].

However, the exact molecular mechanism through which catechins produce all of these beneficial effects still remain to be addressed. There is increasing evidence that the membrane is a potential target for the action of catechins. They alter the properties of phospholipid membranes [9], modify the rigidity of the membrane [10], and change the organization of lipid rafts [11,12]. The concept of the membrane as a simple barrier has evolved to include the membrane as a complex structure with biological functions and an identity intrinsic to the type of cell or disease. In this way, it is possible to look at the mechanism of action of catechins from a lipid and membrane centered perspective [13]. By incorporating into membranes, catechins can alter the conformational dynamics of the membrane, and these changes may have a variety of effects on cellular functions through the indirect modulation of membrane proteins such as receptors, channels, enzymes, and regulatory signals [14]. In this context, the study of the interaction between catechins and membranes is of the utmost importance.

Phosphatidylglycerol is a minor anionic phospholipid component of nearly all natural membranes and has remarkable physical and biochemical characteristics that make it biologically important [15,16]. In eukaryotic cells, phosphatidylglycerol is largely restricted to the mitochondrial membrane, and it is interesting as catechins have been shown to regulate mitochondrial membrane permeability [17] and to protect mitochondria against various insults [18]. Phosphatidylglycerol acts as a lipid signal that promotes early keratinocyte differentiation suppressing skin inflammation [19] and it inhibits Toll-like receptor activation, thereby reducing inflammatory signals [20]. It has been shown that phosphatidylglycerol is able to inhibit inflammatory responses when located in the lung surfactant [21] and in the mitochondria [22], and also to stimulate α-synuclein amyloid formation [23] and to suppress influenza A virus infection [24]. It is important to note that phosphatidylglycerol is a main constituent of virtually all bacterial membranes [25] and thus it has become a good biomimetic model for these membranes [26,27]. This last feature is important in connection with the known antibacterial activity of catechins [28]. It should be highlighted that there are numerous studies that have demonstrated selective toxicity of catechins against different bacterial pathogens, and which have suggested promising use in the treatment of bacterial infections [29,30]. Catechins have been shown to inhibit bacterial toxins, they inhibit the hemolytic activity of *S. aureus* α-toxin preventing the secretion of the toxin by binding to the cytoplasmic membrane and decreasing its permeability, or by blocking signal transduction processes [31]. A mechanism via interaction with the outer cell membrane has been suggested for the inhibitory effect of catechins on enterohemorrhagic Vero toxin from *E. coli* [32]. Catechins also hold promise as biofilm inhibitors [33]. A main mechanism of the antibacterial activity of catechins is the modification of cell membrane [34]. Catechins bind to bacterial cell membrane causing the membrane to burst and release the cytoplasmic content, with subsequent cell death [35], they decrease membrane fluidity that eventually results in membrane breakage [36]. These polyphenols can also inhibit multidrug bacterial efflux pumps restoring the antibacterial activity of antibiotics and acting in synergy with them [37].

**Figure 1:**
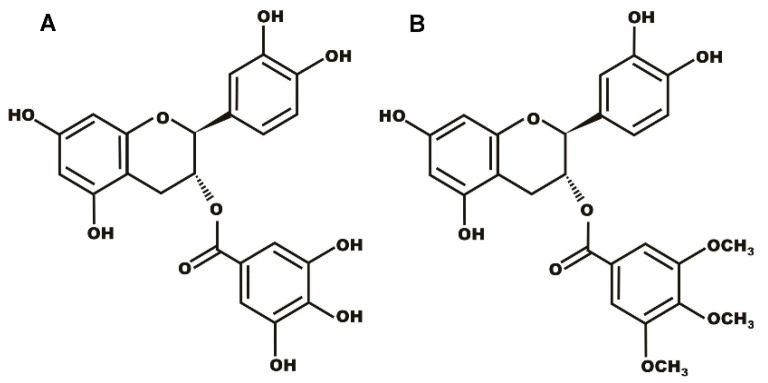
Chemical structure of (**A**) catechin gallate and (**B**) 3,4,5-trimethoxybenzoate of catechin (TMBC).

Regardless of their diverse advantageous characteristics, the therapeutic effect of catechins is restricted as a result of their low stability, poor absorption, limited membrane permeability, and low bioavailability [3,38]. An important strategy to improve the properties of catechins is the structural modification of the catechin molecule through the synthesis of a catechin-based analog in order to obtain more effective, stable, and specific active molecules [5,39]. TMBC (Figure 1) is a structural analog of catechin that has been shown to exert high antiproliferative activity against melanoma and breast cancer cells [40,41]. It has been shown that TMBC perturbs the structural properties of the bilayers composed of phospholipids bearing different polar head groups, including phosphatidylcholine [42], phosphatidylethanolamine [43], and phosphatidylserine [44]. In this work, in order to advance the knowledge of the molecular interaction between catechins and individual membrane phospholipid species, we present a study on the effect of TMBC on the structural and dynamic properties of biomimetic model systems composed of 1,2-dimyristoyl-sn-glycero-3-phospho-(1′-rac-glycerol) (DMPG), by using differential scanning calorimetry (DSC), X-ray diffraction, Fourier transform infrared (FTIR) spectroscopy, and molecular dynamics simulation.

## 2. Results and Discussion

### 2.1. Differential Scanning Calorimetry

**Figure 2:**
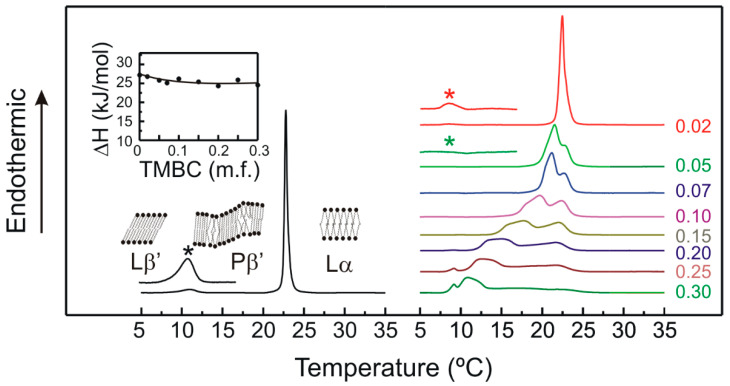
DSC heating thermograms for pure DMPG (left) and DMPG containing TMBC at different concentrations (right). Inset shows the enthalpy change for the main gel to liquid-crystalline phase transition. TMBC mole fraction is expressed on the right side of the thermograms. Asterisks indicate the enlarged region (×7) of the pretransition.

DSC is a straightforward and potent nonperturbing physical technique, which is well appropriate to observe and depict the thermotropic phase transition of membranes [45]. In order to investigate the molecular interaction between TMBC and membranes we used model bilayer systems formed by DMPG. DSC was used to describe the effect of this semisynthetic catechin on the thermotropic properties of this phospholipid. The perturbation exerted by TMBC on the thermotropic phase transition of DMPG is shown in Figure 2.

The heating thermogram corresponding to pure DMPG showed two transitions, a peak to lower temperature starting at 9 °C corresponding to the weakly energetic pretransition from the gel tilted phase (Lβ′) to the gel ripple phase (Pβ′), and a peak to higher temperature starting at 22.3 °C corresponding to the highly energetic main transition from the ripple phase to the liquid-crystalline phase (Lα). These values are in accordance with previous data [46,47]. The presence of TMBC at very low concentrations such as 0.02 mole fraction makes the pretransition broaden and shift to lower temperatures, and the increase of TMBC to 0.05 mole fraction made the pretransition undetectable. There is a possibility that the pretransition had already disappeared and then the phospholipid directly underwent the transition from the gel tilted to the fluid phase. However, it is most probable that the pretransition had already started at such a low temperature that was out of the range under study and that it was so broad that it could not be observed. The presence of increasing concentrations of TMBC produced the broadening of the main transition and the appearance of a second peak with a temperature that was lower when the presence of TMBC in the bilayer was greater. The presence of different peaks in the thermograms, may indicate the presence of different lipid domains in the bilayer. The enthalpy change associated with the main gel to liquid-crystalline phase transition of pure DMPG was determined to be 27.2 kJ/mol. As the inset in Figure 2 shows, the presence of low proportions of TMBC produced a decrease of nearly 10% of the enthalpy change; this value did not decrease further when the concentration of the compound increased above 0.07 mole fraction.

The broadening of the transition and the appearance of additional components, indicate that TMBC incorporates into the phosphatidylglycerol bilayer, where it modifies the organization of the acyl chains of the phospholipid and shifts the phase transition temperature to lower values. It is interesting to note that the ending temperature of the transition did not change with the presence of TMBC; even in the case of the most concentrated sample there was still a portion of the phospholipids that finished its phase transition at the same temperature as the pure phospholipid. Additionally, a new peak emerged at a fixed temperature, near 8 °C, when TMBC reached 0.20 mole fraction. The appearance of a transition peak at a lower and fixed temperature has also been detected in zwitterionic bilayers containing elevated concentrations of TMBC [42,43].

### 2.2. X-ray Diffraction

**Figure 3:**
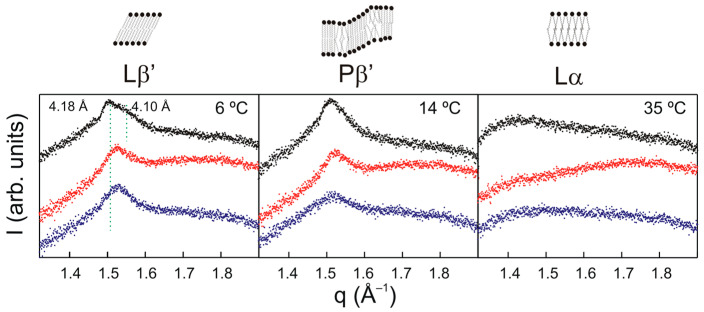
Intensity (arbitrary units lineal scale) vs. scattering vector (*q*) for WAXD profiles of pure DMPG (top black) and DMPG containing TMBC at 0.07 (middle red) and 0.20 mole fraction (bottom blue) at different temperatures.

X-ray diffraction is an acknowledged non-interfering exploratory technique which allows the examination of the overall structural organization of model membranes [48]. We used small- and wide-angle X-ray diffraction (SAXD and WAXD) to address the effect of TMBC on the overall structural properties of DMPG bilayers. Measurements in the wide angle (WAXD) region provide information about the packing of the phospholipid acyl chains. Figure 3 displays the WAXD patterns corresponding to pure DMPG and DMPG containing TMBC. At 6 °C, pure DMPG gave a sharp reflection at 4.18 Å and a broad one at 4.10 Å. This asymmetrical pattern is representative of lipids organized in the Lβ′ phase with orthorhombic packing and the hydrocarbon chains tilted to the membrane surface [49,50]. At 14 °C, pure DMPG showed a symmetrical reflection near 4.16 Å ascribed to the Pβ′ phase in which the hydrocarbon chains were oriented normal to the bilayer plane in a two-dimensional hexagonal lattice [51,52]. Finally, at 35 °C, a diffuse scattering reflection was observed, which is representative of the fluid Lα phase [53].

At 6 °C, in the presence of TMBC, the asymmetric reflection characteristic of the Lβ′ phase was replaced by a symmetric reflection at 4.13 Å corresponding to the Pβ′ phase, which is consistent with the shifting of the pretransition to lower temperatures as observed by DSC (Figure 2). At 14 °C, all the systems displayed the symmetric reflection characteristic of the Pβ′ phase, though the reflection in the presence of TMBC appeared at 4.13 Å instead of 4.16 Å, as is the case for pure DMPG. At 35 °C, all the systems presented the diffuse reflection characteristic of the Lα fluid phase.

**Figure 4:**
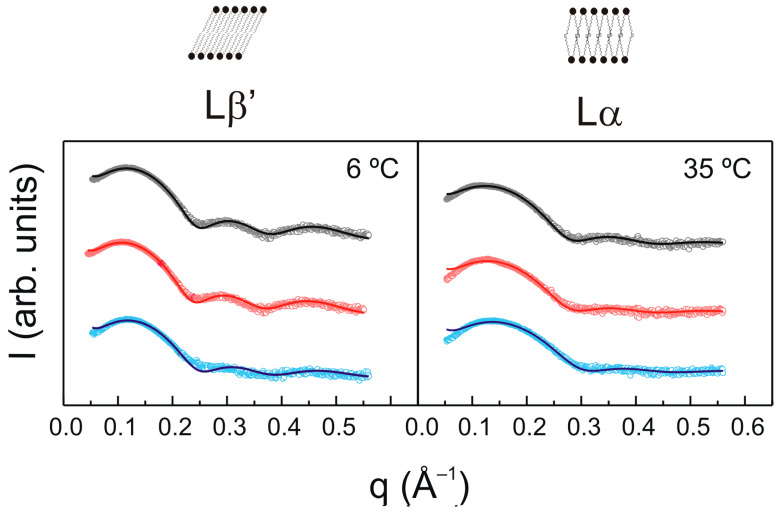
Intensity (arbitrary units log scale) vs. scattering vector (*q*) for SAXD profiles of pure DMPG (top, black) and DMPG containing TMBC at 0.07 (middle, red) and 0.20 mole fraction (bottom, blue) at different temperatures. Solid lines represent the best fit to the experimental patterns using the GAP program.

The SAXD patterns for pure DMPG and DMPG containing TMBC, at different temperatures are shown in Figure 4. All the systems exhibited broad scattering at all temperatures that originated from positionally uncorrelated bilayers. This has been interpreted by the general negative surface charge that drive the formation of positionally uncorrelated bilayers, most likely vesicles with fewer lamellae, because of electrostatic repulsion [47,54]. All systems show a broad bilayer peak around *q* = 0.12 Å^−1^ which arose from the electron density contrast between the bilayer and the solvent, similar to that which has been described for pure DMPG [55].

The analysis of the SAXD patterns using the Global Analysis Program (GAP) enabled us to determine the bilayer thickness (d_B_) of the different systems. We found d_B_ values of 50.16 ± 0.15 Å in the gel phase (6 °C) and 45.20 ± 0.35 Å in the liquid-crystalline phase (35 °C) for pure DMPG, in accordance with previous data [50,55]. The presence of TMBC at 0.07 mole fraction induced a small decrease in the bilayer thickness to 49.23 ± 0.15 Å and 44.80 ± 0.20 Å for the gel and the liquid-crystalline phases. However, the presence of TMBC at 0.2 mole fraction produced a marked decrease in the bilayer thickness, with dB values of 48.16 ± 0.15 Å in the gel phase and 42.60 ± 0.25 Å in the liquid-crystalline phase.

The phospholipid acyl chains were in contact with the hydrophobic parts of integral membrane proteins, and these interactions have been proposed to be crucial for the balanced integration of the protein into the bilayer [56]. Hydrophobic mismatch occurs when the hydrophobic thickness of the membrane does not match with the hydrophobic length of the integral protein. This modification of the membrane properties could be capable of altering the function of lipid-dependent proteins because it could produce changes in the structure of the protein [57]. Considering that integral membrane proteins are involved in crucial cellular processes, the decrease in the bilayer thickness exerted by TMBC might be decisive when considering the hydrophobic mismatch and may have an influence on the mechanism of some of the effects of the catechin. The thinning effect of TMBC was observed at relatively high concentration of the compound. We believe that low concentrations of catechins in the blood stream would correspond to a much greater availability in the membrane fraction and that they may accumulate over time to produce cellular concentrations that are much higher than that observed in serum samples. Moreover, the molar ratios studied in our biomimetic membranes are not necessarily required to be homogeneous in the whole cellular membrane, it would be enough that this TMBC/phospholipid be fulfilled locally in certain part of the membrane. In this respect, the described propensity of TMBC to form enriched domains in the bilayer may help to locally attain higher concentrations of the molecule where it is needed.

**Figure 5:**
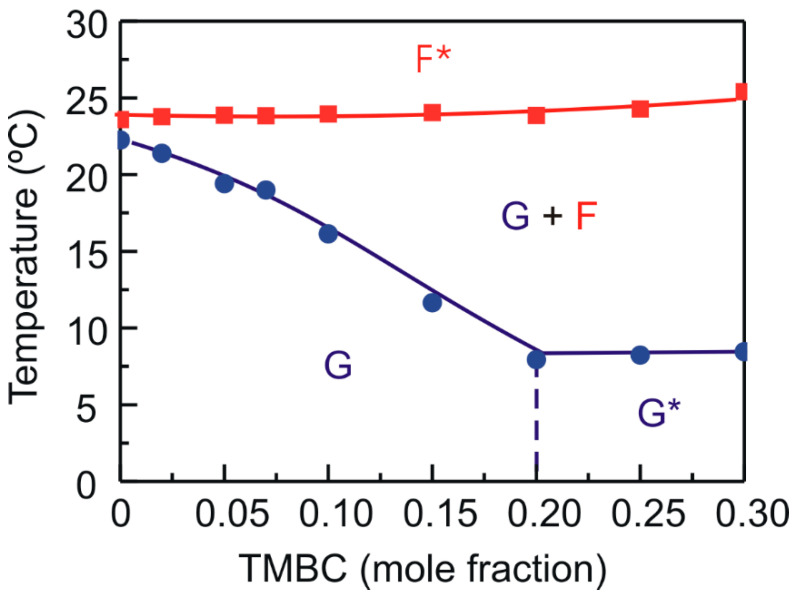
Partial phase diagram for DMPG in DMPG/TMBC mixtures. Circles and squares were obtained from the onset and completion temperatures of the main gel to liquid-crystalline phase transition, respectively. Blue circles, solidus line; red squares, fluidus line. The phase designations are as follows: G, gel phase; F, liquid-crystalline phase (fluid phase); asterisk indicates that immiscible phases are present.

We used the phase transitions temperatures obtained from the DSC measurements and the structural information from the X-ray diffraction experiments to construct the partial phase diagram for the DMPG component in mixtures with TMBC, which are presented in Figure 5. When an incorporated compound in the phospholipid bilayer shows good mixing behavior, i.e., it is miscible with the lipid, the interaction between them will cause the phospholipid transition temperature to change. In this case, the higher the concentration of the compound in the mixture the larger the change in the transition temperature. If the presence of an increasing concentration of a compound in the bilayer does not result in a transition temperature change, i.e., a constant transition temperature is observed, it means that there is always pure phospholipid undergoing transition and suggests that the compound is immiscible with the phospholipid. In this case, an immiscibility was produced with phase separation between the compound and the phospholipid, and with the formation of different domains containing different amounts of compound.

The behavior of the solidus line was different from that of the fluidus line. The solidus line displayed good mixing behavior with its temperature decreasing as more TMBC was present in the bilayer. When a 0.2 mole fraction was reached, the temperature remained constant suggesting the presence of a gel phase immiscibility. This gel phase immiscibility was not observed in mixtures of TMBC and another anionic phospholipid such as phosphatidylserine [44]. The temperature of the fluidus line remained constant in the whole range of TMBC concentrations which suggests that an immiscibility in the liquid-crystalline phase was present. The TMBC-induced fluid-phase immiscibility has not been observed in mixtures of TMBC with any other glycerophospholipids with different polar head groups [42,43,44]. The DMPG system evolves from a gel phase, which shows immiscibility from a certain catechin concentration, to an immiscible fluid phase through a coexistence region which is wider as more TMBC is present in the bilayer. The phase diagram for the TMBC/DMPG mixture seemed to be singular as it showed immiscibility both in the gel and the liquid-crystalline phases.

### 2.3. FTIR Spectroscopy

**Figure 6:**
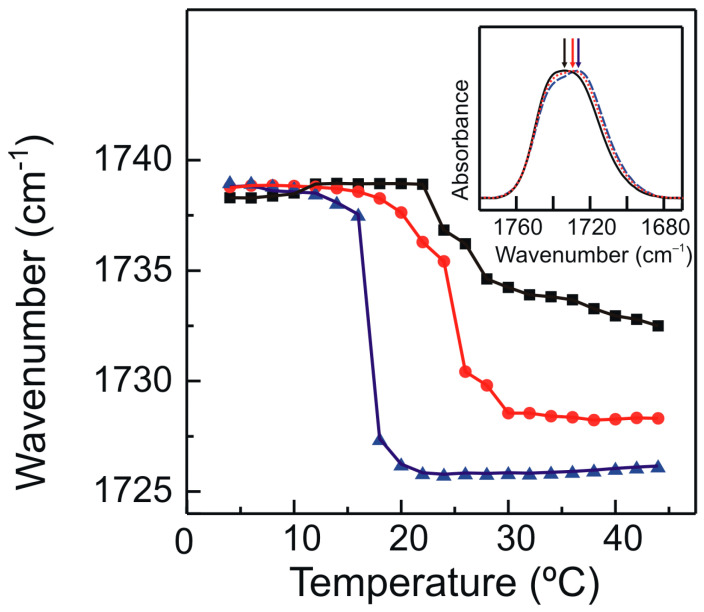
Temperature dependence of the wavenumber of the maximum of the ester carbonyl stretching band, ν (C=O), exhibited by pure DMPG (black squares) and DMPG containing TMBC 0.07 (red circles) and 0.20 (blue triangles) mole fraction. Inset shows the 1780–1670 cm^−1^ spectral region containing the absorption band originating from the ester carbonyl stretching band of pure DMPG (black solid line) and DMPG containing TMBC at 0.07 (red dotted line) and 0.20 (blue dashed line) mole fraction, at 35 °C. Arrows point to the wavenumber of the maximum of each band at this temperature.

Infrared spectroscopy determines the energy transitions between the electronic vibrational levels arising from the absorption of radiation in the infrared spectrum. These vibrational levels are produced by distinctive motions taking place within the different chemical bonds present in the various functional groups of a molecule. The infrared spectrum of phospholipids contains abundant data about both the chemical structure of the molecule and the membrane physical state (chain ordering, phase transition). The gel to liquid-crystalline phase transition of phospholipids goes by apparent changes in the absorption bands originating from moieties in the hydrophobic and interfacial regions of these phospholipid membranes [58]. We used infrared spectroscopy to study the interfacial interaction between the catechin derivative and the bilayer. Figure 6 shows the temperature dependence of the wavenumber of the maximum of the carbonyl stretching band of the infrared spectra corresponding to pure DMPG and DMPG/TMBC systems.

The thermotropic phase changes undergone by phospholipids can be followed by very apparent changes in the contours of the ester carbonyl stretching band, ν (C=O). The features of this absorption band are sensitive to the conformation, hydration state, and the degree and nature of hydrogen-bonding interactions in the polar/apolar interfaces of phospholipids bilayers [59]. A pure DMPG ester carbonyl stretching band was considered to be a summation of two component bands centered near 1742 cm^−1^ and 1728 cm^−1^, and their relative intensities reflect the contribution of a subpopulation of non-hydrogen bonded and hydrogen bonded carbonyl groups [46].

For pure DMPG, the gel to liquid-crystalline phase transition produced a shift in the wavenumber of the maximum of the band to lower wavenumbers. This was due to the increase in intensity of the underlying component band at 1728 cm^−1^. This increase is attributed to a higher amount of hydrogen bonded carbonyl groups that resulted from the increase in the hydration of the polar/apolar interface in the liquid-crystalline phase [60]. The shift in the phase transition to lower temperatures produced by the presence of TMBC can be seen following the wavenumber of the maximum of the carbonyl band illustrated in Figure 6. It is interesting to note that in the liquid-crystalline phase, the presence of increasing concentrations of TMBC produced a shift in the maximum of the carbonyl band to lower wavenumbers, as compared with the pure phospholipid. The latter indicated an increase in the proportion of the hydrogen bonded carbonyl groups, and this could have been due to a direct interaction of TMBC with the interfacial region of the DMPG bilayer or to an indirect mechanism through the TMBC disordering the membrane and increasing the hydration of the carbonyl groups of the phospholipid.

### 2.4. Molecular Dynamics

**Figure 7:**
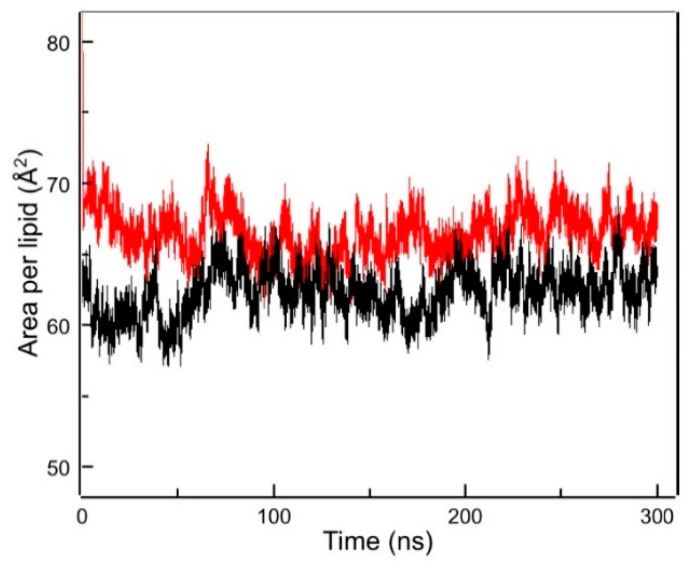
Area per lipid vs. simulated time for pure DMPG (black line) and DMPG containing TMBC (red line). Last 60 ns were used for all MD analysis.

Computer simulation, such as molecular dynamics, has proven to be an important contribution to biophysical research on the physicochemical properties of lipid membranes, as it provides atomic detail of the simulated system [61]. The area per lipid at the membrane aqueous interface is frequently used as a property of the lipid bilayer for validating molecular dynamics simulations and as a proof of convergence [62]. Figure 7 shows the progression of the area per lipid of the simulation runs, where it can be observed that the area per lipid reached convergence and kept constant in the time range used for all the analyses (last 60 ns). The area per lipid was calculated as the area of the x y plane of the simulation box divided by the number of lipids in each leaflet. For the pure DMPG bilayer in the liquid-crystalline phase the area per lipid was 0.63 ± 0.01 nm^2^, in accordance with reported data [63], and in the presence of TMBC this value increased to 0.66 ± 0.01 nm^2^.

The bilayer thickness was computed calculating the phosphorous atoms distance between both leaflets. For pure DMPG, we obtained a bilayer thickness of 3.37 ± 0.07 nm, while in the presence of TMBC we observed a decrease in the bilayer thickness to 3.29 ± 0.07 nm, the latter data being in agreement with the X-ray diffraction experiments discussed above.

The number of hydrogen bonds between the DMPG carbonyl groups and water and the TMBC molecules were measured in the simulation box. The data show that the total number of hydrogen bonds per lipid increased from 202.2 ± 4.6 for pure DMPG to 212.5 ± 5.7 in the presence of TMBC. This increase was mostly due to the new hydrogen bonds established between the carbonyl groups of the phospholipid and the hydroxyl groups of TMBC (7.10 ± 2.15), this being in agreement with the increase in the number of hydrogen bonds determined by the shift of the wavenumber of the maximum of the carbonyl absorption band to lower values as obtained by FTIR (Figure 6).

**Figure 8:**
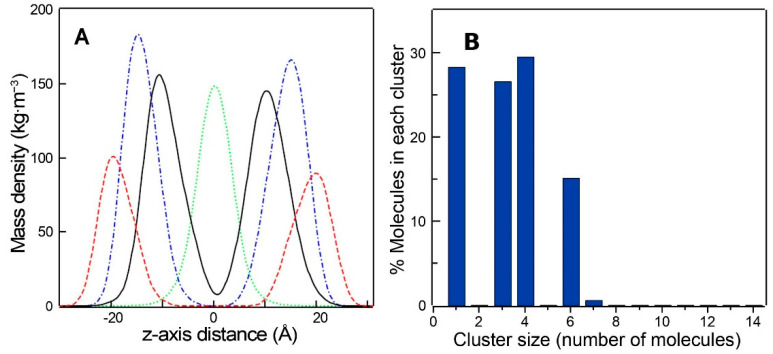
(**A**) Non-symmetrized mass density profiles along the *z*-axis of the simulation box of DMPG/TMBC system at 308 K. TMBC molecule in solid black line, phosphorus atoms in dashed red line, lipid carbonyl groups in blue dash one dot line and lipid terminals methyl carbon atoms in green dotted lines. (**B**) Cluster size distribution of TMBC molecules in the DMPG bilayer.

The mass density profiles of the simulated DMPG bilayer in the presence of TMBC are shown in Figure 8A. The non-symmetrized mass density profiles correspond to the profiles along the *z*-axis of the simulation box over the entire analyzed time trajectory. The lipid phosphorous atoms are included to label the polar head region, the lipid terminal methyl groups to label the center of the membrane and the lipid carbonyl groups to label the position of the hydrogen bonding. The TMBC molecules were mainly distributed across the middle region of each monolayer approaching the carbonyl moiety of DMPG. In the different simulations, TMBC molecules were located at different random starting locations in the lipid phase, but this did not produce differences in the final output of the location of TMBC molecules across the phospholipid bilayer.

The propensity of TMBC to form aggregates was examined by determining the cluster size distribution of TMBC in the bilayer, and it was calculated as the number of TMBC molecules that were found in the analyzed trajectory within a distance of 0.25 nm. Figure 8B shows that the proportion of TMBC molecules present as monomers was 28.30 ± 0.05%, and the rest of TMBC forms aggregates of 3–6 molecules. The proportion of TMBC monomers in the DMPG bilayer was lower than the in case of phosphatidylserine bilayer [44], where more than 50% of TMBC was present as monomers.

In order to gain insight into the differences observed between phosphatidylglycerol and phosphatidylserine systems, we determined the hydrogen bonds of the phospholipid headgroups and TMBC, and the data are presented in Table 1. There were more hydrogen bonds between the headgroups of dimyristoylphosphatidylserine (DMPS) than between those of DMPG, both in the absence and presence of TMBC. In the presence of TMBC, there were more hydrogen bonds between TMBC and the headgroup of DMPG than between TMBC and those of DMPS, probably reflecting the higher availability of DMPG headgroups to form hydrogen bonds with other molecules than the phospholipid itself. Table 1 also shows the hydrogen bonds formed between different TMBC molecules. It was observed that TMBC formed more intermolecular hydrogen bonds in the DMPG bilayer than in the DMPS one. These data contribute to explaining the different effects exerted by TMBC on the different phospholipids, and the higher cluster formation in the case of DMPG. The presence of these different clusters in the liquid-crystalline bilayer may explain the presence of different domains in the DSC thermograms (Figure 2) and the strong tendency of TMBC to form clusters in DMPG systems may explain the presence of immiscibility in the liquid-crystalline phase (Figure 5).

**Figure 9:**
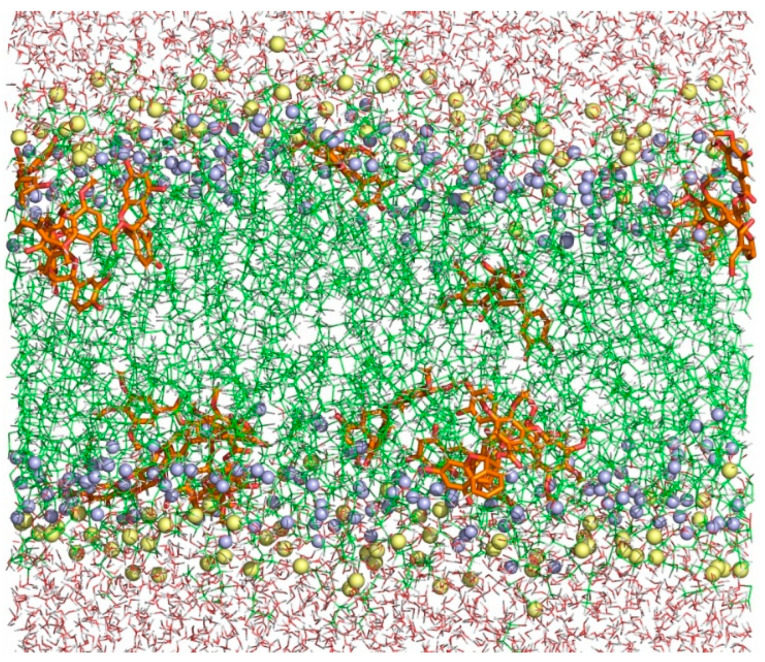
Final snapshot of the simulation box at 308 K of DMPG + TMBC. Water molecules are shown with purple lines, TMBC with orange and red sticks, DMPG with green lines, lipid carbonyl groups with blue spheres, and phosphorous atoms with yellow spheres.

Figure 9 shows a snapshot of the simulation box at 308 K of the DMPG/TMBC mixture where the location of TMBC in the bilayer can be observed. This is the expected location for TMBC considering the experimental results reported above. The location near the center of each monolayer allows TMBC to perturb the gel to the liquid-crystalline phase transition and the proximity to the carbonyl region of DMPG enables the catechin to interfere with the hydrogen bonding pattern of the interfacial region of the bilayer.

## 3. Materials and Methods

### 3.1. Materials

1,2-Dimyristoyl-sn-glycero-3-phospho-(1′-rac-glycerol) (sodium salt, dimyristoylphosphatidylglycerol, DMPG) (>99% TLC) was purchased from Avanti Polar Lipids Inc. (Birmingham, AL, USA). Phosphorous analysis was used to determine the phospholipid concentration [64]. (-)-Catechin and 3,4,5-trimethoxybenzoyl chloride were purchased from Sigma Chemical Co. (Madrid, Spain), and TMBC was synthesized from catechin as detailed previously [40]. All other reagents were of the highest purity available.

The ratios between TMBC and phospholipid used in this study were similar to those commonly used in previous studies on the interaction between catechins and membranes [9,65] and ranged from low TMBC concentration in the membrane (0.02 mole fraction) to high concentration of TMBC in the membrane (0.30 mole fraction). TMBC is a synthetically modified catechin and no data is yet available concerning physiological concentrations. However, a correlation between these ratios and the concentrations of TMBC exhibiting anticancer activity can be established. It has been recently reported for an epithelial cell line that the phospholipid content was around 2 μg Pi/10^6^ cells [66]. If we assume that the phospholipid content in melanoma cells is similar to this value and we consider the IC_50_ of 1.5 μM for TMBC in melanoma cells [40], it renders that under the cell culture conditions (number of cells and volume of the medium) the TMBC/phospholipid ratio in the antiproliferative studies was around 0.3 mole fraction. Hence, the molar fraction used in our study was in the range of those expressing biological activity.

### 3.2. Differential Scanning Calorimetry

Samples for DSC were prepared by drying organic solvent solutions containing convenient quantities of DMPG and TMBC, and forming model bilayer vesicles in 150 mM NaCl, 0.1 mM EDTA, 10 mM Hepes, and pH 7.4 buffer, essentially as described previously [42]. In the case of DMPG, due to the electrostatic repulsion that is generated by the negative surface charge of the phospholipid, this thin-film hydration and manual agitation method produced vesicles with fewer lamellae. The experiments were carried out in a MicroCal PEAQ-DSC calorimeter (Malvern Panalytical, Malvern, UK) at 1.5 mM final phospholipid concentration, and heating scan rate of 60 °C h^−1^. Data were analyzed using ORIGIN v7 (Northampton, MA, USA). Areas under the thermograms were used to determine the enthalpy change of the transitions. The onset and completion temperatures for each transition peak were obtained from the heating thermograms taken at the points of intersection of the tangents to the leading edges of the endotherms and the baselines, and were plotted as a function of the mole fraction of TMBC to construct a partial phase diagram.

### 3.3. X-ray Diffraction

Samples containing 10 μmol of DMPG and a convenient amount of TMBC were prepared similarly to those described for DSC. The suspensions were centrifuged for 30 min at 12,000 rpm in order to obtain concentrated samples to ensure that the diffraction intensities were high enough to be usable. Pellets were placed in a steel holder and were measured in a modified Kratky compact camera (MBraum-Graz-Optical Systems, Graz, Austria). Nickel-filtered Cu Kα X-rays were generated by a Philips PW3830 X-ray Generator operating at 50 kV and 30 mA. SAXD and WAXD were accomplished at the same time essentially as previously described [31]. The *q* (scattering vector) range covered (*q* = 4π sin θ/λ; where 2θ is the scattering angle and λ = 1.54 Å the selected X-ray wavelength) was between 0.05 and 0.6 Å^−1^ for SAXD and from 1.32 to 1.95 Å^−1^ for WAXD. Background corrected SAXD data were analyzed using the program GAP (Global Analysis Program) written by Prof. Georg Pabst (University of Graz, Austria) and obtained from the author [67,68]. In this program, the membrane is modeled as a sheet of infinite lateral extent with an electron density profile that is taken to be given by the summation of two headgroup Gaussians of width σ_H_ and position ± Z_H_, as well as a hydrocarbon chain Gaussian of width σ_C_ and negative amplitude located at the center of the bilayer at Z = 0. For randomly oriented bilayers that exhibit no positional correlations, such as DMPG vesicles, the scattered intensity is given by

$$I\left(q\right)=\frac{{\left|F\left(q\right)\right|}^{2}}{q^{2}}$$

where the form factor *F*(*q*) is the Fourier transform of the electron density profile [40]. This program allows the membrane thickness to be retrieved, d_B_ = 2 (Z_H_ + 2σ_H_) from a full *q*-range analysis of the SAXD patterns [69]. The width σ_H_ of the Gaussian peak applied to model electron density profile of the head group region was fixed to 3 Å. Data were presented as mean values ± S.E. (*n* = 3).

### 3.4. Infrared Spectroscopy

Samples for the infrared measurements containing 10 μmol of DMPG and convenient amount of TMBC were formed in 75 μL amounts of the same buffer prepared in D_2_O as described above. Infrared spectra were collected in a Nicolet 6700 FTIR spectrometer (Thermo Fisher Scientific, Madison, WI, USA) essentially as described previously [42]. Spectra were analyzed using the software Grams (Galactic Industries, Salem, NH, USA).

### 3.5. Molecular Dynamics

The molecular structure of TMBC was constructed from the chemical structure of (-)-catechin gallate obtained from the PubChem Substance and Compound data base [70] through the identifier number 6419835. All molecular dynamics simulations were carried out using GROMACS 5.0.7 and 2018.1 [71] with the aid of the Computational Service of the Universidad de Murcia (Spain).

CHARMM36 force field parameters for DMPG, TMBC, water, chloride and sodium ions were obtained from CHARMM-GUI [72,73,74]. Packmol software [75] was used to build the initial membrane structures formed by two leaflets oriented normal to the *z*-axis. The bilayer membrane was built with 128 molecules of DMPG with and without 14 molecules of TMBC, with a water layer containing a total of 6400 water molecules (TIP3 model), 144 sodium ions, and 144 chloride ions. DMPG and TMBC were randomly distributed in each phospholipid layer keeping the DMPG molecules oriented normal to z-x plane, All systems were simulated using the NpT-ensemble at 308 K constant average temperature. Pressure was controlled semi-isotropically at 1 bar of pressure and 4.5 × 10^−5^ bar^−1^ of compressibility. The cutoffs for van der Waals and short-range electrostatic interactions were set to 1.2 nm, and a force switch function was applied between 1.0 and 1.2 nm. Equilibration was undertaken for 3 ns using the V-rescale temperature coupling method, and Berendsen pressure coupling method [76]. Equilibration was followed by production runs of 300 ns using the Nose-Hoover thermostat [77] and the Parrinello-Rahman barostat [78]. Graphical representation and inspection of all molecular structures were carried out with PyMOL 2.3.0 [79]. The last 60 ns of the production run were used for the analysis by using Gromacs tools. The used timestep was 2 fs. The reported hydrogen bonds were calculated with the corresponding GROMACS tool with distances between donor and acceptor of ≤0.35 nm and an angle between hydrogen-donor and donor-acceptor of 30°. The mass density profile was calculated with the corresponding GROMACS tool, assuming no symmetry between both monolayers and centered to the origin Z = 0. Three replica simulations for the DMPG/TMBC system were performed. In the different simulations the TMBC molecules were at different random starting locations in the lipid phase. Data were presented as mean values ± S.E. (*n* = 3).

## 4. Conclusions

We investigated the molecular interactions between TMBC and biomimetic bilayer membranes of DMPG employing a mixed experimental and computational approach. The DSC experiments showed that TMBC incorporated into the DMPG bilayer where it was able to perturb the gel to liquid-crystalline phase transition, giving rise to the formation of immiscible lateral domains both in the gel and the liquid-crystalline phase. X-ray diffraction measurements indicated that TMBC promoted the formation of the ripple gel phase in DMPG, and it was able to produce a decrease in the bilayer thickness. The FTIR experiments illustrated how TMBC established an alteration of the hydrogen bonding pattern in the interfacial region of the bilayer. The experimental results support the simulation data, where a decrease in the bilayer thickness and an increase in the number of hydrogen bonds were determined. Simulation experiments locate the semisynthetic catechin molecules as monomers and small clusters in the middle region of the DMPG acyl chain palisade reaching the interfacial carbonyl region in agreement with the effect observed by experimental techniques. We believe that the observed interactions between TMBC and DMPG generate physical disturbances that might alter membrane function, and may help to discern the mechanism of action of the increasing list of biological action of catechins.

## Figures and Tables

**Table 1:** Hydrogen bonds of the phospholipid polar headgroups and TMBC. The dimyristoylphosphatidylserine (DMPS) data presented were obtained from previously reported simulations [44].

		Hydrogen Bonds	S.E. (*n* = 3)
DMPS	DMPS-DMPS	193.59	5.21
DMPS + TMBC	DMPS-DMPS	185.13	6.26
	TMBC-DMPS	13.51	0.88
	TMBC-TMBC	1.26	0.48
DMPG	DMPG-DMPG	147.89	4.36
DMPG + TMBC	DMPG-DMPG	145.49	4.39
	TMBC-DMPG	16.55	1.13
	TMBC-TMBC	4.95	0.83

## Data Availability

Not applicable.
